# Symbiotic microbiome *Staphylococcus epidermidis* restricts IL-33 production in allergic nasal epithelium via limiting the cellular necroptosis

**DOI:** 10.1186/s12866-023-02898-7

**Published:** 2023-05-26

**Authors:** Yung Jin Jeon, Chan Hee Gil, Jina Won, Ara Jo, Hyun Jik Kim

**Affiliations:** 1 Department of Otorhinolaryngology, Gyeongsang National University Hospital, Jinju, Republic of Korea; 2 Department of Otorhinolaryngology, Seoul National University College of Medicine, Seoul, Republic of Korea; 3 Sensory Organ Research Institute, Seoul National University Medical Research Center, Seoul, Republic of Korea; 4 Department of Otorhinolaryngology-Head and Neck Surgery, Seoul National University College of Medicine, 103, Daehak-ro, Jongno-gu, Seoul, 03080 Republic of Korea

**Keywords:** *Staphylococcus epidermidis*, Allergic rhinitis, Interleukin-33, Necroptosis, Nasal epithelium

## Abstract

**Background:**

Allergic rhinitis (AR) is characterized by airway inflammation in nasal mucosa from inhaled allergens and interleukin (IL)-33 is the potent inducer of Th2 inflammation in allergic nasal epithelium. *Staphylococcus epidermidis* is one of the most abundant colonizers of the healthy human nasal mucosa and might impact the allergen-induced inflammatory responses in the nasal epithelium. Thus, we sought to characterize the mechanism of *S. epidermidis* regulating Th2 inflammation and IL-33 production in AR nasal mucosa.

**Results:**

The AR symptoms were alleviated and eosinophilic infiltration, serum IgE levels, and Th2 cytokines were significantly decreased in OVA-sensitized AR mice in response to human nasal commensal *S. epidermidis*. The inoculation of *S. epidermidis* to normal human nasal epithelial cells reduced IL-33 and GATA3 transcriptions and also reduced IL-33 and GATA3 expression in AR nasal epithelial (ARNE) cells and the nasal mucosa of AR mice. Our data exhibited that the cellular necroptosis of ARNE cells might be involved in IL-33 production and inoculation of *S. epidermidis* decreased the phosphorylation of necroptosis enzymes in ARNE cells, which was related to the reduction of IL-33 production.

**Conclusions:**

We present that human nasal commensal *S. epidermidis* reduces allergic inflammation by suppressing IL-33 production in nasal epithelium. Our findings indicate that *S. epidermidis* serves a role in blocking allergen-induced cellular necroptosis in allergic nasal epithelium which might be a key mechanism of reduction of IL-33 and Th2 inflammation.

**Supplementary Information:**

The online version contains supplementary material available at 10.1186/s12866-023-02898-7.

## Background

Allergic rhinitis (AR) is initiated according to inhalant allergen exposure at the nasal epithelium and the inhaled allergen-provoked inflammatory process begins with increased secretion of epithelial cell-derived cytokines. This allergen-specific immune response is thought to arise from an imbalance in T helper (Th)1-Th2 immune regulation that results in increased levels of Th2 cytokines [1, 2]. Actually, nasal epithelial cells which are exposed to external allergens induce Th2 inflammatory responses and allergic inflammation occurs in the upper airway or AR subjects [3]. The allergen-mediated inflammatory immune response begins with increased secretion of epithelial cell-derived cytokines which provide critical signals to innate and adaptive cell populations related to Th2 inflammation [4, 5]. Interleukin (IL)-33, which is dominantly produced by the airway epithelium, is a key epithelial-derived cytokine involved in the pathogenesis of allergic airway diseases and provides an essential axis for rapid allergic immune responses [6]. Remarkable progress has recently been achieved to identify the biological function of IL-33 in the nasal epithelium of AR and the potential value of IL-33 as a novel therapeutic target of AR at the nasal epithelium level is strongly suggested [7–10]. Therefore, we sought to understand the mechanism by which IL-33 is secreted from epithelial cells to induce an allergic reaction.

As stated before, IL-33 production is stimulated by tissue damage and its various cleavage sites by caspases regulating extracellular activity [8, 10]. Recently, the necroptosis of epithelial cells has been suggested as a release mechanism of IL-33, which is driven not by apoptotic caspases but by receptor-interacting protein kinase 3 (RIPK3)/mixed lineage kinase-like protein (MLKL)-dependent cell death pathway, control IL-33 release [11]. However, studies on whether inhibition of cellular necroptosis can reduce the secretion of IL-33 from the nasal epithelium and regulate AR inflammation are still lacking.

Studies of the nasal symbiotic microbiome and host-to-host communication incorporate environmental cues in the nasal mucosa that reflect the effects of immune responses and specific symbiotic microbiome-mediated defenses from external pathogens. [12]. In the nasal mucosa, inhaled allergens first encounter the host immune system, and the microbial properties of nasal mucus have a direct impact on the mechanism of the initial allergic reaction in the nasal epithelium [12–14]. Insights into the microbiota and dysbiosis of allergic nasal mucosa provide basic information regarding the relationship between susceptibility to allergens and allergic inflammation, including the stimulation of epithelial cell-derived Th2 cytokines such as IL-33. [6, 15]. Recent studies have determined how the nasal symbiotic microbiota modulates sensitization tolerance to inhaled allergens in asthma [16–18]. Intriguingly, our knowledge of microbial composition in allergic nasal mucus is limited, and a mechanistic consideration of immunomodulatory properties of the nasal microbiome to inhaled allergens has not been comprehensively performed in AR. Previously, we have shown that the composition of the nasal commensal microbiota differs between healthy volunteers and AR patients [6]. *Staphylococcus epidermidi*s is the most dominant bacterial strain in both healthy volunteers and AR patients, and the distribution of *S. epidermidis* significantly is increased in AR patients compared to healthy volunteers [19]. We hypothesized that this alternated structure of nasal commensal microbiota in AR, which is known as dysbiosis, provides supporting information regarding susceptibility to inhaled allergens and their association with allergic inflammatory response, including Th2 cytokines induction through control of epithelial cell-derived cytokines such as IL-33.

The purpose of this study was to show the distinctive role of the nasal microbiome *S. epidermidis*, a major symbiont in healthy nasal mucus, and the potential impact on the reduction of Th2 inflammation through decreased cellular necroptosis and subsequent IL-33 release in nasal epithelium.

## Results

### Administration with human nasal commensal ***S. epidermidis*** suppresses allergic inflammation in vivo

**Fig. 1:**
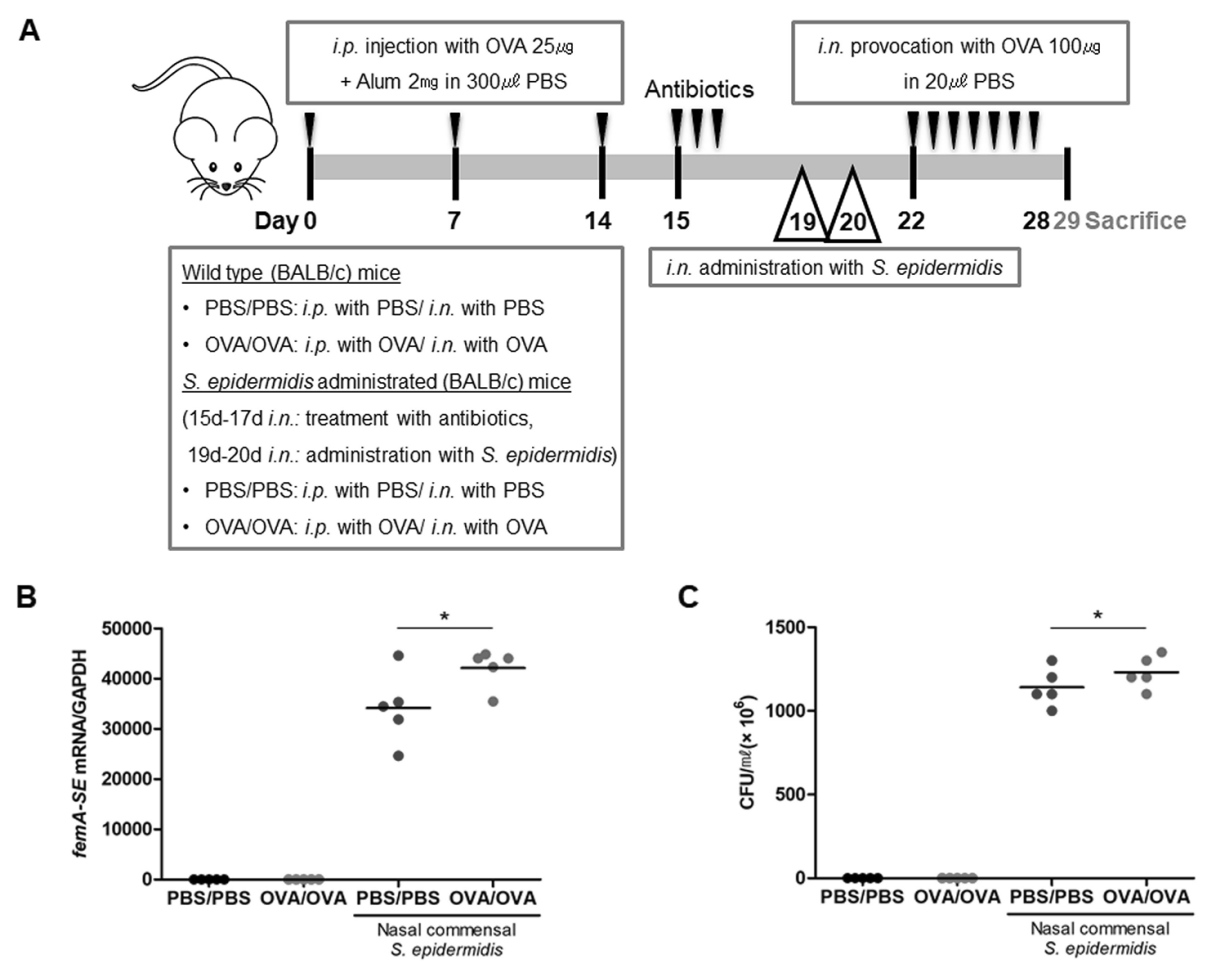
**Experimental protocol of intranasal inoculation with*S. epidermidis*in OVA-driven AR mouse model**. (**A**) The experimental protocol for the development of AR mouse using BALB/C. Mice were sensitized by intraperitoneal injection of ovalbumin mixed with aluminum hydroxide on days 0, 7, and 14. Daily OVA intranasal challenge was performed from days 22 to 28 (OVA/OVA). Human nasal *S. epidermidis* (3.2 × 10^6^ CFU/30 µl PBS) was inoculated at indicated time points (day 19, 20). (**B**) mRNA expression of femA gene, specific for *S. epidermidis* and normalized to cellular GAPDH transcript levels, were monitored by real-time PCR. (**C**) Colony counts of nasal lavage fluid were performed

**Fig. 2:**
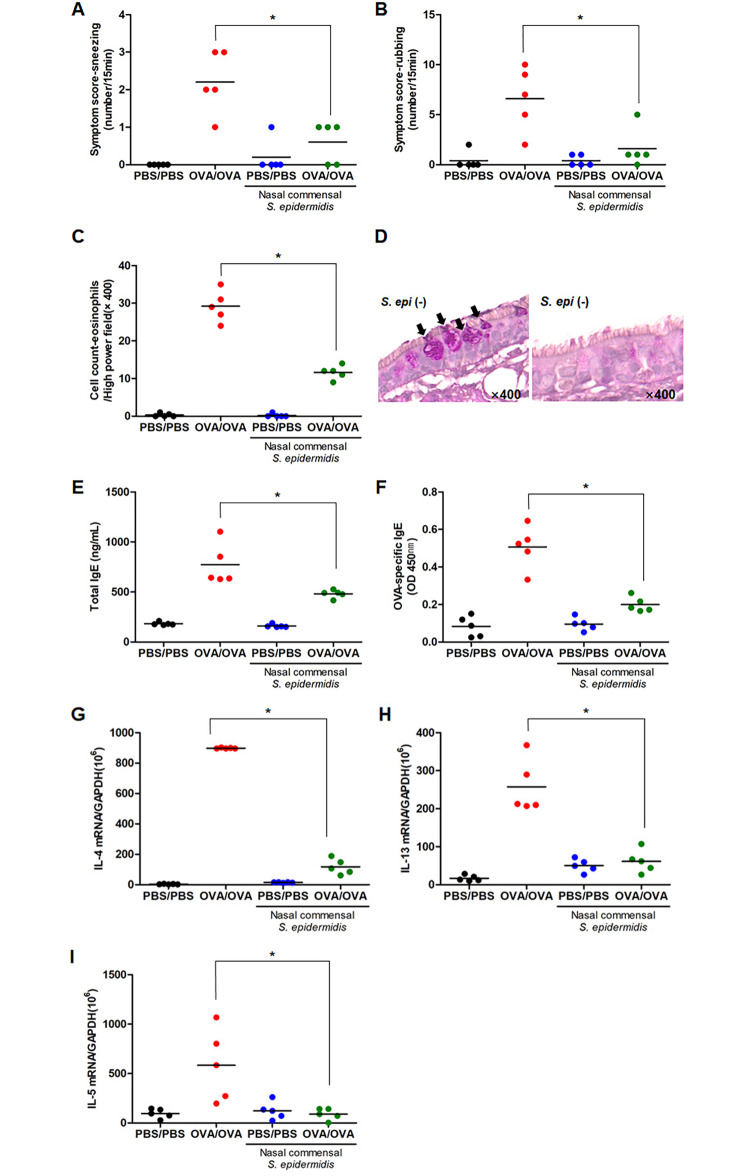
**The alteration of allergic inflammation and transcription of Th2 cytokines in nasal mucosa of OVA-driven AR mouse in response to*S. epidermidis***. Human nasal *S. epidermidis* (3.2 × 10^6^ CFU/30 µl PBS) was inoculated at indicated time points to the nose of OVA-sensitized BALB/C mice. Frequencies of sneezing (**A**) and rubbing (**B**) events were assessed over a 15 min period after OVA provocation. Histologic findings in nasal mucosa of each group (×400 magnification) with Sirius red staining for eosinophils (**C**) and Periodic Acid-Schiff (PAS) staining for secretory cells (**D**). Serum levels of total IgE (**E**) and OVA-specific IgE (**F**) were significantly lower in the nasal mucosa of AR-OVA with inoculation of *S. epidermidis* than in AR-OVA mice (N = 5). The mRNA expressions of *IL-4* (**G**), *IL-5* (**H**), *IL-13* (**I**) were determined using real-time PCR. Results are presented as mean ± standard deviation (SD) (N = 5). *p < 0.05

First of all, we sought to explore whether the human nasal commensal *S. epidermidis* exhibited anti-allergic properties using an in vivo murine model of AR. BALB/C mice were sensitized and challenged with OVA (AR-OVA, N = 5) and they were also inoculated with human nasal commensal *S. epidermidis* for 2 days (day 19, 20) following nasal microbiota depletion (day 15, 16, 17) using 30 µl of an antibiotic cocktail (vancomycin, neomycin, ampicillin, and metronidazole) (Fig. 1A). Then, *femA-SE* mRNA expression and colony count of *S. epidermidis* were measured in *vivo* nasal mucosa to confirm effective colonization of the commensal *S. epidermidis* in WT and AR-OVA mice after administration. The results revealed that *femA-SE* mRNA expression and colony count from NAL fluid were significantly increased in WT (1.15 × 10^9^ ± 0.71 × 10^9^ CFU/mℓ) and AR-OVA mice (1.85 × 10^9^ ± 3.54 × 10^8^ CFU/mℓ) with the intranasal inoculation of *S. epidermidis* than in WT and AR-OVA mice without *S. epidermidis* inoculation (Fig. 1B and C). The difference in allergic symptoms of in vivo OVA-AR mice was observed depending on the inoculation of *S. epidermidis* (Fig. 2A and B). The scores for sneezing and rubbing were 2.20 ± 0.84 and 6.60 ± 3.21 in AR-OVA mice and were significantly reduced in AR-OVA mice with the *S. epidermidis* administration (0.60 ± 0.55, *p* = 0.009 and 1.60 ± 1.95, *p* = 0.022, respectively). Eosinophils and secretory cells of nasal mucosal tissue were detected by Sirius red and PAS staining respectively. The absolute eosinophil count in the submucosa of AR-OVA mice administrated with *S. epidermidis* was significantly lower than in AR-OVA mice (29.25 ± 4.79 and 11.50 ± 2.08, *p* = 0.002, Fig. 2C). Fewer PAS-stained secretory cells were observed in nasal mucosa of *S. epidermidis*-administrated AR-OVA mice (Fig. 2D). Serum levels of total IgE (Fig. 2E) and OVA-specific IgE (Fig. 2F) were significantly decreased in AR-OVA mice after inoculation with *S. epidermidis*, respectively. The real-time PCR data showed that intranasal inoculation of *S. epidermidis* markedly reduced mRNA levels of *IL-4*, *IL-5*, and *IL-13* in the nasal mucosa of AR-OVA mice (Fig. 2G-I). These findings revealed that the human nasal commensal *S. epidermidis* suppresses allergic inflammation in AR in vivo model and reduces transcription of Th2 cytokines in the nasal mucosa.

### Nasal commensal ***S. epidermidis*** suppresses IL-33-dependent Th2 immune responses in allergic nasal epithelium

**Fig. 3:**
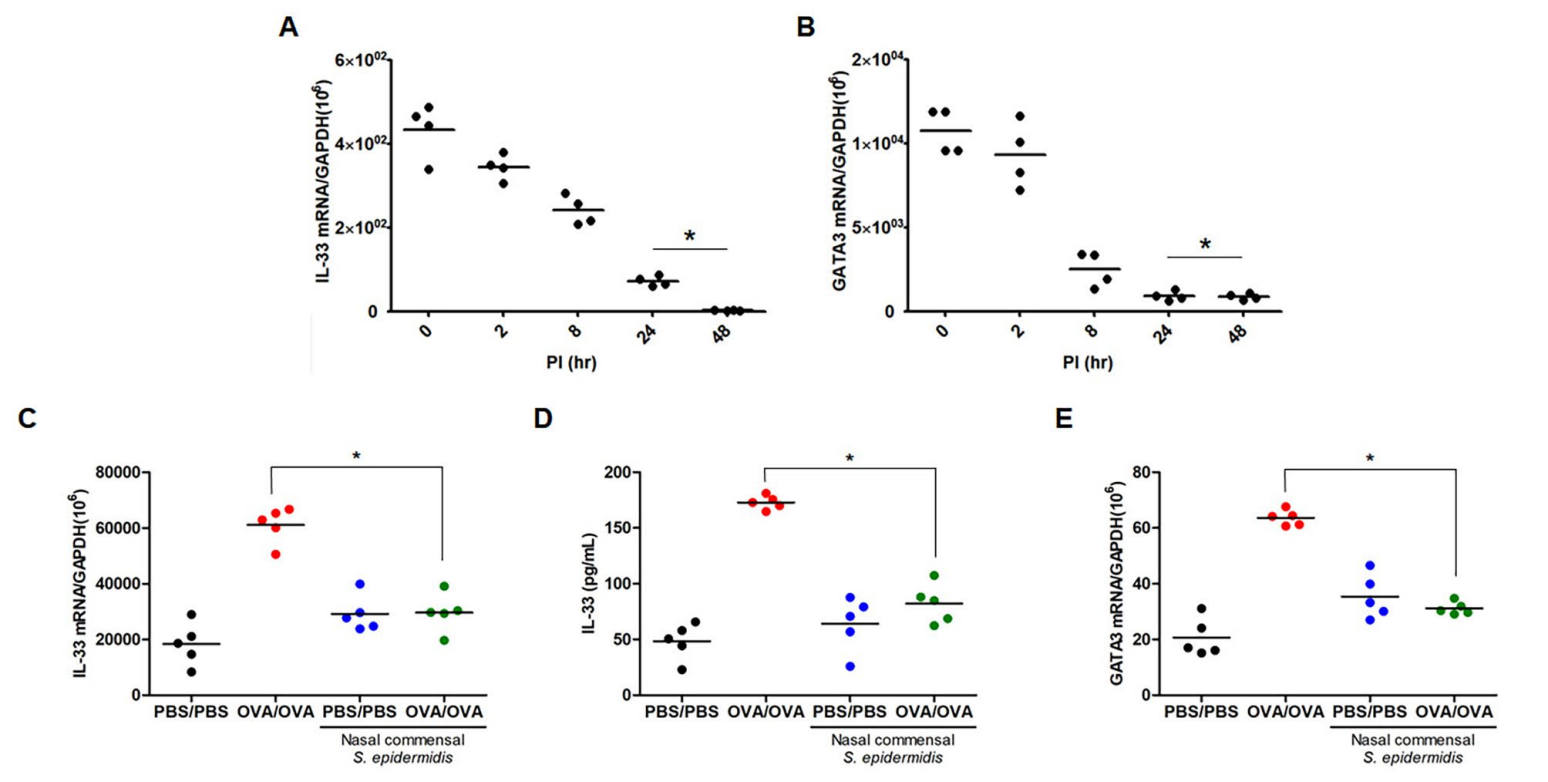
**Human nasal commensal*S. epidermidis*promoted the reduction of IL-33 production in the nasal epithelium**. ARNE cells from four AR subjects were inoculated with human nasal commensal, *S. epidermidis* at a multiplicity of infection (MOI) of 0.25. Levels of mRNAs encoding epithelial cell-derived cytokines including interleukin including IL-33 (**A**) and GATA3 (**B**) which is transcription factor involved in Th2 cell differentiation were monitored by real-time PCR. Results are presented as mean ± SD values from four independent experiments. *p < 0.05 vs. mock-infected ARNE cells. Wild type mice (PBS/PBS) and AR mice (OVA/OVA) were inoculated with human nasal *S. epidermidis* (3.2 × 10^6^ CFU/30 µl PBS) at indicated time points. The mRNA expression of *IL-33* (**C**) was measured by real-time PCR and IL-33 protein concentration (**D**) secreted from nasal mucosa were measured by ELISA using nasal lavage fluid. The mRNA expression of *GATA3* (**E**) was also measured using real-time PCR. Results are presented as mean ± standard deviation (SD) (N = 5). *p < 0.05

To determine the response of epithelial-derived Th2 cytokines in the allergic nasal epithelium, the human nasal *S. epidermidis* was inoculated into human ARNE cells for 2 dpi and the transcriptions of IL-33 and TSLP were measured using real-time PCR. The data showed that transcription of IL-33 was markedly attenuated after 1 dpi in ARNE cells after inoculation with *S. epidermidis*, which likely reduced *GATA3* transcription (Fig. 3A and B). In addition, the IL-33 mRNA level (Fig. 3C) and secreted IL-33 and GATA3 protein concentrations (Fig. 3D and E) were significantly reduced in the nasal mucosa of AR-OVA mice in response to inoculation with human nasal *S. epidermidis*. However, TSLP mRNA expression and secreted protein concentration were slightly decreased in AR-OVA mice with the intranasal inoculation of *S. epidermidis* (*p* = 0.164 and *p* = 0.374, respectively) (supplementary Fig. S1A and B). These findings demonstrated that *S. epidermidis*-regulated anti-allergic immune responses might be involved in IL-33 downregulation of the nasal mucosa of in vivo AR model.

### Nasal commensal ***S. epidermidis*** reduces IL-33 release by cellular necroptosis in ARNE cells

**Fig. 4:**
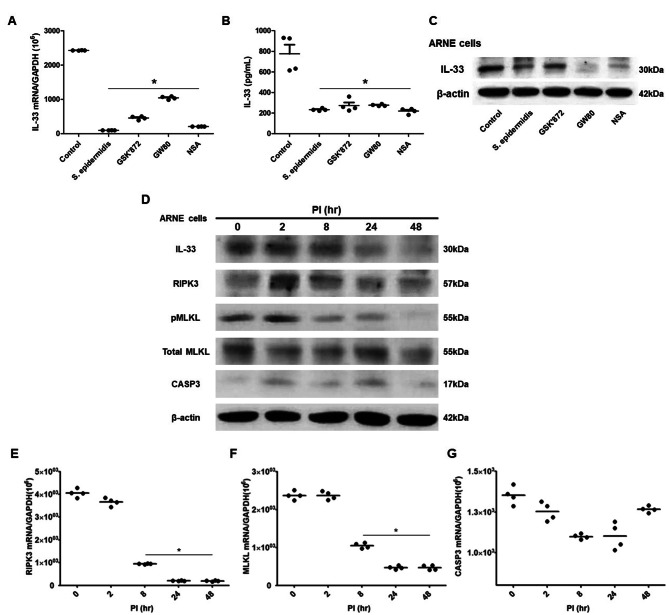
***S. epidermidis*reduces IL-33 production via suppression of RIPK3/MLKL-dependent necroptosis and is independent of apoptosis in allergic nasal epithelium**. ARNE cells from four AR subjects were inoculated with human nasal commensal, *S. epidermidis* at a multiplicity of infection (MOI) of 0.25. The mRNA expression of IL-33 (**A**) was measured by real-time PCR, IL-33 protein concentration (**B**) secreted from ARNE cells was measured by ELISA using supernatants, and intracellular IL-33 protein expression (**C**) was also measured. after treatment of necroptosis inhibitors. Key molecules of necroptosis including RIPK3 (cropped blot) and MLKL and apoptosis including caspase3 were monitored by Western blot analysis (**D**). The mRNA expression of *RIPK3* (**E**), and *MLKL* (**F**), and caspase3 (**G**) was measured by real-time PCR. Results are presented as mean ± SD values from four independent experiments. *p < 0.05 vs. mock-infected ARNE cells

Lastly, we sought to understand whether *S. epidermidis* reduces IL-33 production by regulating necroptosis of nasal epithelial cells. First, we treated GSK’872 (RIPK3 inhibitor), GW80 (necroptosis inhibitor), and NS (MLKL inhibitor) to ARNE cells for suppression of cellular necroptosis and both transcription and protein expression of IL-33 were determined using real-time PCR, ELISA and western blot analysis. The results revealed that transcription of IL-33, extracellular secreted IL-33 concentration and intracellular IL-33 protein expression were markedly attenuated both after inoculation with *S. epidermidis* and after treatment of necroptosis inhibitors in ARNE cells (Fig. 4A-C).

The regulatory kinetics of the full IL-33, RIPK3, pMLKL, and CASP3 in response to *S. epidermidis* were studied in ARNE cells to assess the necroptosis pathway and both real-time PCR and western blot analysis were performed using cell lysates at the indicated time points after inoculation of *S. epidermidis*. The results of western blot analysis showed that full-length IL-33, total form of RIPK3 and phosphorylation of MLKL protein were reduced at 24 hpi and 48 hpi in *S. epidermidis*-inoculated ARNE cells and particularly, RIPK3 protein expression was significantly decreased from 8 hpi (Fig. 4D). The real-time PCR results revealed that the mRNA levels of *RIPK3* (Fig. 4E) and *MLKL* (Fig. 4F) genes were significantly attenuated from 8 hpi and reduced expression were maintained until 48 hpi in *S. epidermidis*-inoculated ARNE cells. However, there was no significant change in CASP3 protein expression and mRNA levels after inoculation of *S. epidermidis* (Fig. 4D and G). The result elucidated that RIPK3/MLKL-dependent necroptosis pathway might be involved in the suppression of IL-33 expression and human nasal commensal *S. epidermidis* reduced IL-33 production from ARNE cells vis suppression of RIPK3/MLKL-dependent necroptosis.

## Discussion

Our study revealed that human nasal commensal *S epidermidis* reduced the allergic inflammation in ARNE cells and an AR murine model through regulating IL-33 release from nasal epithelium. Following inoculation of *S. epidermidis*, the cellular necroptosis could be limited in AR nasal epithelium, and subsequent IL-33 production as an alarmin signaling was suppressed, resulting in the improvement of Th2 inflammation and AR-related pathologic findings. Our findings imply that intranasal inoculation of *S. epidermidis* might be a potent therapeutic option for controlling Th2 inflammation in nasal mucosa with distinctive mechanism from the existing medication of AR.

Recent studies have highlighted the interaction between the symbiotic microbiota and the host immune system, which play an important role in host immune homeostasis by evolutionary pressures on the host immune system in contrast to the pathogen [20–22]. There is also growing evidence that a microbiome community resides in the human nasal mucus and exhibits the potential crosstalk between the nasal microbiome and Th2 allergic inflammation in the upper airway [23, 24]. In agreement with our previous works, colonization by *Staphylococcus species* is highly increased in the nasal mucus of subjects with AR and particularly, inoculation of isolated nasal commensal *S. aureus* suppresses Th2 cytokine-dependent allergic inflammation by regulating epithelial cell-derived cytokines including IL-33, in in vitro and in vivo AR models [6]. We determined that additional studies whether the most dominant commensal *S. epidermidis* in healthy nasal mucus mediates anti-allergic immune responses to define the clear mechanism of host–bacterial commensalism. Actually, we found that inoculation of *S. epidermidis* was involved in the alteration of transcription profiles including T-cell activation and Th2 immune responses in NHNE cells. In particular, inoculation of human nasal commensal *S. epidermidis* to cultured ARNE cells and in vivo AR mice reduced allergic inflammation significantly by suppressing the transcription, translation and extracellular release of IL-33 from nasal epithelial cells.

It is noteworthy that IL-33 is a key molecule to induce allergic inflammation in the airway and known that functions as an alarm signal inducing Th2 cytokines [25]. Moreover, IL-33 acts as a proinflammatory cytokine in various immune responses by promoting local inflammatory responses through the recruitment of Th2 inflammatory effectors [25, 26]. There are three epithelial cell-derived cytokines such as IL-25, TSLP, and IL-33 known to mediate Th2 inflammation and IL-33 has been identified to initiate type 2 responses in asthma in response to the clinically relevant allergens including HDM [27]. Previously we also found that IL-33 expression was preferentially elevated in both primary cultured ARNE cells and nasal mucosa of OVA-driven AR mouse model [6, 15]. The current study showed that human nasal commensal *S. epidermidis* significantly attenuated IL-33 expression in both in vitro and in vivo AR models. These results indicate that *S. epidermidis* from healthy human nasal mucus provides a promising therapeutic route of AR by limiting IL-33 production in nasal epithelium and understanding of how IL-33 activity can be regulated by *S. epidermidis* will be necessary for controlling Th2 immune responses.

Up to date, several studies suggested that IL-33 might be released from the extracellular space following cellular damage or mechanical stress caused by allergens or infection [27, 28], and that IL-33 be released into plasma following necroptosis, which was characterized by regulated cell death pathway representing features of apoptosis and necrosis [29, 30]. We also found that inhaled allergens-caused cellular necroptosis might be required for IL-33 release in nasal epithelium. Inoculation of nasal commensal *S. epidermidis* could be involved in the restriction of cellular necroptosis of nasal epithelium. Our bulk-seq data revealed attenuation of RIPK3 gene expression in nasal epithelium following inoculation of *S. epidermidis* (supplementary Fig. S2 and Table S1). Both translation of RIPK3 and phosphorylation of MLKL were all significantly reduced in AR nasal epithelium in response to *S. epidermidis* independent of cellular apoptosis. Based on these findings, we estimate that inoculation of *S. epidermidis* results in reduced IL-33 production in the nasal epithelium and thus may impede Th2 inflammation in the nasal mucosa.

A major difficulty in understanding *S. epidermidis*-downregulated Th2 inflammation was that *S. epidermidis* did not limit the secretion of TSLP, which was also thought to be related to cellular damage of nasal epithelial cells as like IL-33. It can be inferred that TSLP has a distinctive mechanism of transcription and translation from IL-33, and its secretion is thought to be induced by a mechanism independent of cellular necroptosis upon exposure to antigen in nasal epithelium. Another limited point of this study is that we just nasally implanted human nasal commensal *S. epidermidis* in in vivo model and lacked data on interactions with other species of microbiota. Actually, *S. epidermidis* exists with other nasal microorganisms in nasal mucus and does not produce pathogenic determinates depending on sustain beneficial microbial environment [31]. Therefore, further experiments will be needed to demonstrate the alternative function of *S. epidermidis* in whole nasal commensal microbiota and the regulatory influence on additional Th2 cytokines. We acknowledge that limiting the production of IL-33 may not be the only way to regulate allergic inflammation in AR, our study focused to investigate the role and mechanism of *S. epidermidis* in suppressing IL-33 production in nasal epithelium. The balance between Th1 and Th2 immunity plays an important role in the development and regulation of allergic responses [32]. We used primary cultured human allergic rhinitis nasal epithelial cells, and there was a limitation in the experimental method to evaluate the immune response of lymphocytes such as T cells. Further investigation into the role of *S. epidermidis* in regulating Th1 cytokines and type 1 inflammatory responses in AR.

## Conclusions

We described that the human nasal commensal *S. epidermidis* reduces IL-33-dependent Th2 inflammation, thereby preventing the pathologic findings of AR and inoculation of *S. epidermidis* was able to reduce IL-33 expression via RIPK3/pMLKL dependent necroptotic cell death. This intimate association of *S. epidermidis* and the amplification of cellular necroptosis-induced IL-33 secretion could potentially benefit the AR through the downregulation of IL-33 at the level of the nasal epithelium.

## Materials and methods

### Participant recruitment

We recruited 8 subjects referred to the Department of Otorhinolaryngology primarily for septoturbinoplasty in Seoul National University Hospital (Seoul, Korea) and Gyeongsang National University Hospital (Jinju, Korea) between September 2020 and January 2021. The Institutional Review Board of Seoul National University College of Medicine (No. 1709-049-883) and Gyeongsang National University Hospital (No. 2019-05-004) provided approval for this study and all participants provided written informed consent. All of them underwent a multi-allergen simultaneous test (MAST) to detect allergens and specific IgE levels to confirm whether subjects experienced atopic traits (Table 1). AR is defined based on the presence of allergen-specific IgE and documentation of the relationship between allergens and typical symptoms such as runny nose, stuffy nose, itching, and/or sneezing [5]. All participants did not have any history of smoking, asthma, other sinonasal diseases, or taking intranasal or systemic corticosteroids before sampling. Four subjects who did not have AR participated in nasal mucosal swabs to isolate nasal commensal *S. epidermidis*. In addition, intranasal mucosal brushing was done around the middle turbinate for the culture of normal (NHNE) and AR nasal epithelial cells (ARNE) in 4 healthy subjects and 4 AR patients during septoturbioplasty under general anesthesia.

**Table 1:** Demographics of AR patients included in this study

Subjects number	Sex	Age	Allergy test	Total IgE	Positive allergen	Sampling
1	M	31	MAST	92	-	Nasal swab† and mucosa‡
2	F	25	MAST	95	-	Nasal swab and mucosa
3	F	21	MAST	67	-	Nasal swab and mucosa
4	M	20	MAST	87	-	Nasal swab and mucosa
5	M	32	MAST	162	Dp 4, Df 4	Nasal mucosa
6	M	20	MAST	146	Df 4, Mugwort 4, Cat 4	Nasal mucosa
7	M	19	MAST	142	Dp 4, Df 4	Nasal mucosa
8	F	28	MAST	128	Dp 4, Df 3, Dog 4	Nasal mucosa

Abbreviations: MAST, Multi-allergens simultaneous test; Dp, *Dermatophagoides pteronyssinus*; Df, *Dermatophagoides farina*

†Nasal swab: sampling for isolation of nasal microbiome

‡ Nasal mucosa: sampling for culture of nasal epithelium cells (NHNE cells: Subject 3,4,5, ARNE cells: subject 6,7,8)

### Characterization and isolation of S. epidermidis in human nasal mucus

Mucus from the middle turbinate of healthy subjects was collected individually using sterile 3 M Quick swabs (3 M Microbiology Products, St. Paul, MN, USA) from four subjects using a rigid 0-degree endoscope in an operating room. For bacterial colony isolation, the mucus was plated on Lysogeny Broth (LB) plates. After two days of incubation, bacterial colonies were obtained from the LB plates, and the species of each colony was identified using GS-FLX 454 pyrosequencing and 16 S rRNA gene amplification, as described previously [19].

### Cell culture and inoculation with human nasal commensal S. epidermidis

NHNE and ARNE cells were cultured from four healthy subjects and four AR patients (Table 1) using an air-liquid interface (ALI) method [33]. Human nasal commensal *S. epidermidis* was inoculated with NHNE and ARNE cells as previously described [19]. Briefly, the isolated commensal *S. epidermidis* was plated and grown overnight at 37 °C on LB plates. A single colony of *S. epidermidis* was grown overnight at 37 °C in 1 ml of LB liquid medium using an incubation shaker. The mixture of commensal *S. epidermidis* culture was diluted with the non-antibiotic feeding medium to adjust the amount to 300 µl and inoculated into the apical compartment of each NHNE and ARNE cell culture well. NHNE and ARNE cells were incubated for 0, 2, 8, 24, and 48 h at 37 °C in 5% CO_2_.

### Bulk *RNA sequencing* (bulk-seq)

NovaSeq 6000 platforms (Illumina, San Diego, CA, USA), and preliminary sequencing results were converted to FASTQ files using the Cell Ranger pipeline (10× Genomics). We followed the 10× Genomics standard sequence protocol by trimming the barcode and unique molecular identifier end to 26 bp and the mRNA end to 98 bp. Then, the FASTQ files were aligned to the human reference genome (GRCh38). Subsequently, Cell Ranger was applied for preliminary data analysis to generate a file that contained a barcode table, a gene table, and a gene-expression matrix. We used WinSeurat version 2.1 (Ebiogen Inc., Seoul, Korea), based on Seurat version 3, for quality control, analysis, and exploration of bulk-seq data. Data mining and graphic visualization were performed using ExDEGA (Ebiogen Inc.). Gene Ontology (GO) enrichment was evaluated using EnrichR analyses based on the cutoff criteria (adjusted *p* < 0.05, fold change ≥ or < 1.5 and normalized data (log2) ≥ or < 2.0). The original bulk-seq data of the NHNE cells with *S. epidermidis* inoculation are available (data accessible at NCBI Gene Expression Omnibus database, accession GSE167509).

### Murine inoculation model with human nasal commensal S. epidermidis

Animal experiments were approved by the Institutional Animal Care and Use Committees of Gyeongsang National University (No. GNU-201,007-M0065) and the research methods were carried out by the approved guidelines. Five-week-old male wild-type (WT) BALB/C mice (Orient, Busan, Korea) were maintained under specific-pathogen-free conditions, and all mice were housed in a temperature-controlled environment with a 12-hour dark/light cycle. Mice were divided into four groups depending on *S. epidermidis* administration and OVA sensitization, which were the negative control group (WT), AR group (AR-OVA), *S. epidermidis*-administrated WT mice group, and *S. epidermidis*-administrated AR-OVA mice group. AR-OVA mice were sensitized and challenged with OVA as previously described [6]. Briefly, BALB/C mice were sensitized by intraperitoneal injection of 25 ug OVA mixed with 2mg alum on Day 0, 7, and 14 and then challenged by intranasal treatment of 100μg OVA for 7 consecutive days, from Day 22 to Day 28. At the end of the protocol, mice were euthanized by intramuscular injection of a high dose of a mixture of 10 mg/kg xylazine (Bayer, Puteaux, France) and 5 mg/kg ketamine (Merial, Lyon, France). If no heartbeat was detected, death was confirmed. When the mice were euthanized by injection, cervical dislocation was also performed to confirm that the mice were dead. Nasal symptoms such as sneezing and rubbing for 15 min were measured and total and OVA-specific IgE levels were analyzed using a serum sample. Nasal lavage (NAL) fluid was obtained from the nasal cavity by lavaging with 200 µl 0.5mM ethylene diamine tetraacetic acid (EDTA) in phosphate-buffered saline (PBS). The NAL fluid was used for absolute colony count and enzyme-linked immunosorbent assay for measuring secreted protein concentration. Nasal tissue was harvested for real-time PCR and histologic analysis.

### Real-time PCR

Human nasal commensal *S. epidermidis* colonization was monitored using real-time PCR to assess factors that were essential for methicillin-resistant (*femA*) gene expression specific for *S. epidermidis*. The *S. epidermidis* mRNA was monitored using real-time PCR for the *femA* gene with forward and reverse primers and probe 5’-CAACTCGATGCAAATCAGCAA-3’, 5’-GAACCGCATAGCTCCCTGC-3’, and 5′-JOE-TACTACGCTGGTGGAACTTCAAATCGTTATCG-BHQ1-3′, respectively. Levels of transcripts encoding human *IL-33* and *GATA3* and mouse *IL-4, IL-5, IL-13, GATA3, IL-33, TSLP*, *MLKL*, and *RIPK3* were determined using real-time PCR. All primers were purchased from Applied Biosystems (Foster City, CA, USA).

### Western blot

Human primary IL-33 antibody was purchased from R&D Systems (AF3625; Minneapolis, MN, USA). Human primary antibodies for RIPK3 (PA5-19956), phospho-MLKL (Ser358, PA5-105678), total MLKL (PA5-34733), and CASP3 (9H19L2) were purchased from Invitrogen (Waltham, MA, USA).

### Absolute colony count

Bacterial samples were serially diluted 10-fold with phosphate-buffered saline (PBS). Next, 10 µl of each diluted sample was plated onto an LB agar plate and incubated for 24 h at 37 °C. The number of *S. epidermidis* colonies growing on the agar surface was counted manually [6, 36]. Bacterial growth was reported based on the colony-forming units (CFUs) for each sample.

### Serum levels of total and OVA-specific IgE

Serum samples were collected from mice at the time of sacrifice. Serum samples were stored at − 80 °C until further use. Serum levels of total IgE and OVA-specific IgE were measured by enzyme-linked immunosorbent assay (ELISA) as described previously [6]. The total IgE levels were analyzed by coating the plates overnight with anti-mouse IgE capture monoclonal antibody (mAb) (BD PharMingen, San Jose, CA, USA) at 4 °C. The plate was washed with PBST (PBS containing 0.05% Tween-20) three times and nonspecific antigen–antibody reactions were blocked by incubation with 300 µL of 3% bovine serum albumin (BSA) per well for 1 h at room temperature. Serum samples were added to the 96-well plates along with purified mouse IgE isotype (BD PharMingen) used as a standard, and the plates were incubated for 3 h at 4 °C. The total IgE was detected by adding anti-mouse IgE-horseradish peroxidase (HRP) (#1130-055, Southern Biotechnology, Birmingham, AL, USA) to the plates. The OVA-specific IgE was analyzed by coating the plates with 1 mg/mL OVA at 4 °C overnight. The plates were washed three times with PBST, and nonspecific antigen–antibody reactions were blocked by incubation with 300 µL of 3% BSA per well for 1 h at room temperature. Serum samples were added to the plates and incubated for 2 h at room temperature. The OVA-specific IgE were detected by incubating each plate with biotin rat anti-mouse IgE (BD PharMingen). The plates were then washed three times and incubated with Streptavidin-HRP (BD PharMingen) for 30 min at room temperature. The reactions were developed using 3,3′,5,5′-tetramethylbenzidine (TMB) peroxidase substrate (Seracare life science, Milllford, MA, USA) and terminated by adding 1 M HCl. Optical density (OD) was measured in a microplate reader at 450 nm.

### Histologic analysis

Mice heads were fixed in 10% formalin overnight at room temperature, decalcified, and embedded in paraffin blocks. Paraffin-embedded samples were cut into 4 μm-thick sections, deparaffinized in xylene and dehydrated through a series of graded ethanol solutions. Prepared slides of mice heads stained with hematoxylin and eosin (H&E) for inflammatory cell counting, Sirius red stain for eosinophil counting under a microscope (high power field, ×400 magnification) in 4 fields of the nasal septal mucosa, and Periodic Acid-Schiff (PAS) stain to indicate secretory cells of the nasal septal mucosa [6].

### Quantification of secreted cytokines

Secreted human IL-33 (DY3625B) and mouse IL-33 (DY3626) were quantified using the DuoSet® ELISA kit from R&D Systems according to the manufacturer’s instructions.

### Statistical analyses

The analyzed data were expressed as the mean ± standard deviation (SD) of 3 independent experiments, and at least 3 independent experiments were performed using primary cultured cells from each donor. We compared the groups with the Mann-Whitney U test to compare results between negative and positive controls, and treatment groups and positive control. We assessed the differences between treatment groups by one-way analysis of variance (ANOVA) accompanied with a post-hoc test. We considered that a *p*-value < 0.05 was statistically significant. We performed all statistical analyses were using SPSS 25.0 software (IBM, Chicago, IL, USA).

## Electronic supplementary material

Below is the link to the electronic supplementary material.

Additional file 1: Supplementary Figures and Table

Additional file 2: The original and unprocessed blot images Fig. 4C and D

## Data Availability

All data generated or analyzed during this study are included in this published article and its supplementary information files.

## List of abbreviations

AR: Allergic rhinitis
Th: T helper
IL-33: Interleukin-33
RIPK3: Receptor-interacting protein kinase 3
MLKL: Mixed lineage kinase-like protein
NHNE: Normal human nasal epithelium
ARNE: Allergic rhinitis nasal epithelium
TSLP: Thymic stromal lymphopoietin
