# Correction: Symbiotic microbiome *Staphylococcus epidermidis* restricts IL-33 production in allergic nasal epithelium via limiting the cellular necroptosis

**DOI:** 10.1186/s12866-023-02912-y

**Published:** 2023-06-14

**Authors:** Yung Jin Jeon, Chan Hee Gil, Jina Won, Ara Jo, Hyun Jik Kim

**Affiliations:** 1 Department of Otorhinolaryngology, Gyeongsang National University Hospital, Jinju, Republic of Korea; 2 Department of Otorhinolaryngology, Seoul National University College of Medicine, Seoul, Republic of Korea; 3 Sensory Organ Research Institute, Seoul National University Medical Research Center, Seoul, Republic of Korea; 4 Department of Otorhinolaryngology-Head and Neck Surgery, Seoul National University College of Medicine, 103, Daehak-ro, Jongno-gu, Seoul, 03080 Republic of Korea

**Correction: BMC Microbiol 23, 154 (2023)**

**https://doi.org/10.1186/s12866-023-02898-7**

Following the publication of the original article [1], the authors identified an error in the Supplementary Information section as stated below.

The Additional Supplementary File 2 is an outdated version that was not updated with the final revision. However, the uploaded file for Additional Supplementary File 3 represents the correct and final version that should be included as Additional Supplementary File 2. Additional Supplementary File 3 has been removed, and the Additional Supplementary File 2 has now been updated accordingly. We would like to apologize for this error and any inconvenience it may have caused.

The original article has been corrected.
