# Supplementary material for: Symbiotic microbiome Staphylococcus epidermidis restricts IL-33 production in allergic nasal epithelium via limiting the cellular necroptosis
Source: BMC Microbiol. 2023 May 26;23:154. doi: 10.1186/s12866-023-02898-7 (PMC10214541; doi:10.1186/s12866-023-02898-7)
Supplement: Supplementary file 1 — Additional file 1: Supplementary Figures and Table [file 12866_2023_2898_MOESM1_ESM.pdf]

Fig. S1. The production of TSLP in AR in vivo model depending of S. epidermidis inoculation. Wild type mice (PBS/PBS) and AR mice (OVA/OVA) were inoculated with human nasal *S. epidermidis* (3.2×10^6 CFU/30 µl PBS) at indicated time points. (A) The mRNA expression of TSLP was measured by real-time PCR and (B) IL-33 protein level secreted from nasal mucosa were measured by ELISA using nasal lavage fluid. Results are presented as mean ± standard deviation (SD) (N = 5). *$p < 0.05$.

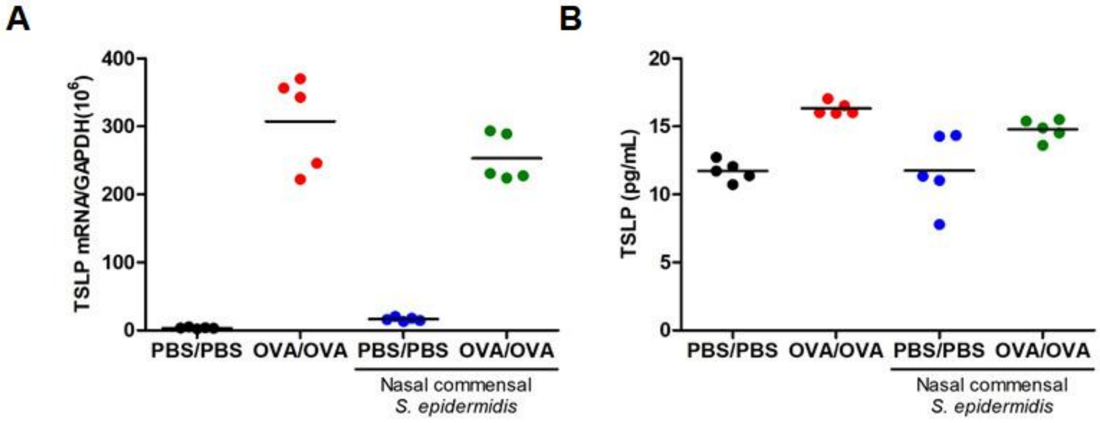

Fig. S2. Integration analysis of RNA sequencing data from commensal *S. epidermidis*-inoculated human nasal epithelial cell culture. NHNE cells from four healthy volunteers were inoculated with human nasal *S. epidermidis* at an MOI of 0.25 and bulk RNA sequencing was performed to determine differentially expressed genes (DEGs) depending on *S. epidermidis* inoculation. (A) Gene Ontology (GO) functional analysis of differentially expressed genes of biological process. The top 20 GO terms of biological processes were plotted according to their *p*-values ranking. (B) Volcano plot of differentially expressed genes. The differentially expressed genes with FDR < 0.05 and log 2 (fold change) >1 are shown. The numbers of downregulated genes (blue) and upregulated genes (yellow) are shown on the top.

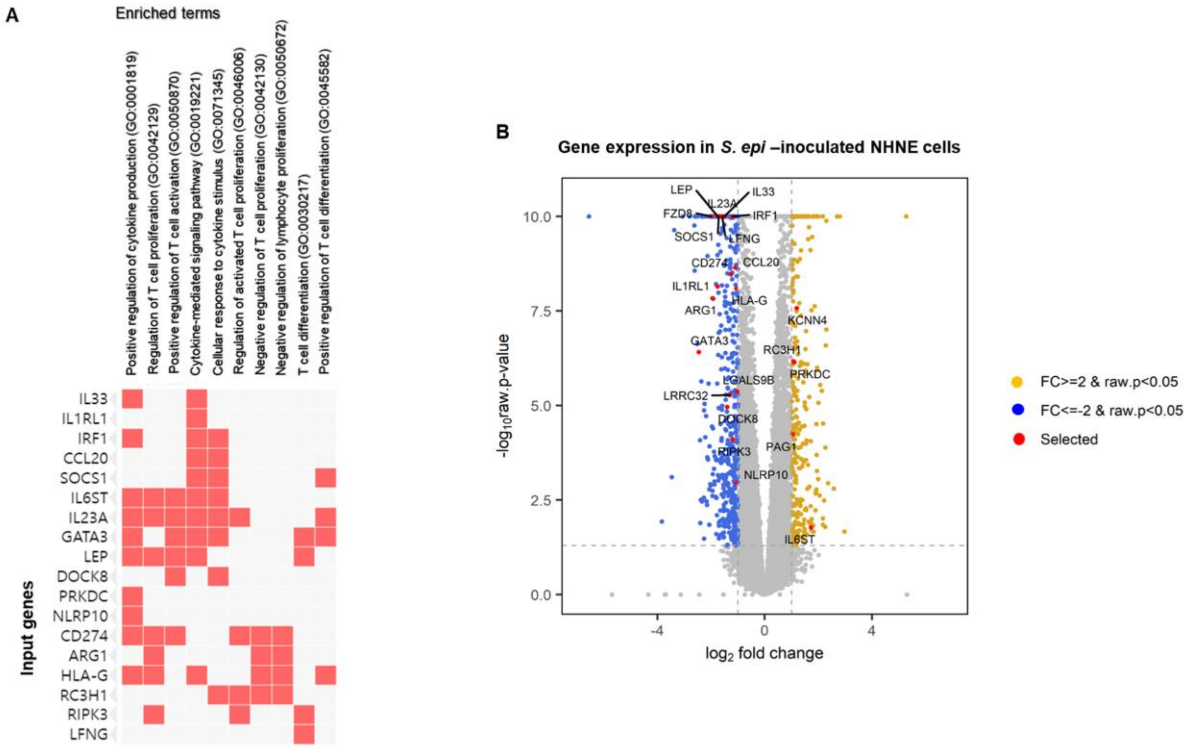

Table S1. Top 10 significant GO biological processes.

Term	*p*-value	Overlap genes
Positive regulation of cytokine production (GO:00019819)	1.27E-12	GBP5.CD274.IL33.CCBE1.CSF2.PRKDC.GATA3.HLA-G.TRIM6.RGCC. NIRP10.IL23A.LEP.IRF1.PDE4B.CHI3L1.FFAR2.IL6ST.TLR5.TLR3
Cellular response to cytokine stimulus (GO:0071345)	2.41E-12	GBP5.CEF2.PNPT1.CCL22.CCL20.IL1R2.DOCK8.RC3H1.GATA3. CBL.MMP9.IL1F10.TRIM6.SOCS1.IL23A.IRF1.IL2RB.ANKRD1.CHI3L1.VIM.SOX9.IL6ST.GBP1
Cytokine-mediated signaling pathway (GO:0019221)	1.01E-11	CSF2.PELI2.IFIT5.GATA3.CBL.SAMHD1.IL1RL1.SOCS1.GBP1.IL33.CCL22. CCL20.IL1R2.HLA-G,MMP9.IL17RB.IL1F10.IL23A.LEP.IRF1.IL2RB.VIM.IL6ST. MAP3K14.IL17C
Regulation of interleukin-8 production (GO:0032677)	1.96E-07	NLRP10.C5AR2.LEP.OTUD7B.CHI3L1.FFAR2.TLR5.TLR3
Regulation of inflammatory response (GO:0050727)	5.52E-07	IL33.NLRP10.IK23A.NAPEPLD.S100A12.FFAR2.GATA3.MMP9. VAMP2.TLR3.BRD4
Neutrophil	9.09E-07	RAB3A.RAB5C.DDX3X.SERPINA1.CALML5.ATG1.

degranulation (GO:0043312)		KRT1.IQGAP2. MMP9.OSCAR.GLIPR1.DOK3.LRG1.S100A12.CHI3L1.VAMP2
Positive regulation of intracellular signal transduction (GO:1902533)	1.01E-06	DDX3X.PELI2.IFIT5.GATA3.FGF1.CBL.TRIM6.IL23A.LEP.REL. S100A12.CHI3L1.SOX9.MAP3K14.IKBKE.TLR3.BRD4
Neutrophil activation involved in immune response (GO:0002283)	1.01E-06	RAB3A.RAB5C.DDX3X.SERPINA1.CALML5.ARG1.KRT1.IQGAP2. MMP9.OSCAR.GLIPR1.DOK3.LRG1.S100A12.CHI3L1.VAMP2
Neutrophil-mediated immunity (GO:0002446)	1.10E-06	RAB3A.RAB5C.DDX3X.SERPINA1.CALML5.ARG1.KRT1.IQGAP2. MMP9.OSCAR.GLIPR1.DOK3.LRG1.S100A12.CHI3L1.VAMP2
Regulation of T cell proliferation (GO:0042129)	1.88E-06	CD274.RIPK3.ARG1.IL23A.LEP.IL6ST.HLA-G
