# Supplementary material for: Symbiotic microbiome Staphylococcus epidermidis restricts IL-33 production in allergic nasal epithelium via limiting the cellular necroptosis
Source: BMC Microbiol. 2023 May 26;23:154. doi: 10.1186/s12866-023-02898-7 (PMC10214541; doi:10.1186/s12866-023-02898-7)

The original and unprocessed blot images of Figure 4C.

ARNE cells
(IL-33)

Control S. epi. GSK'872 GW80 NSA

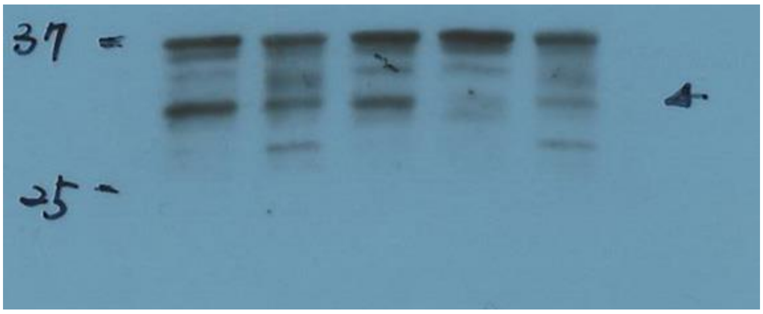

ARNE cells
(β-actin)

Control S. epi. GSK'872 GW80 NSA

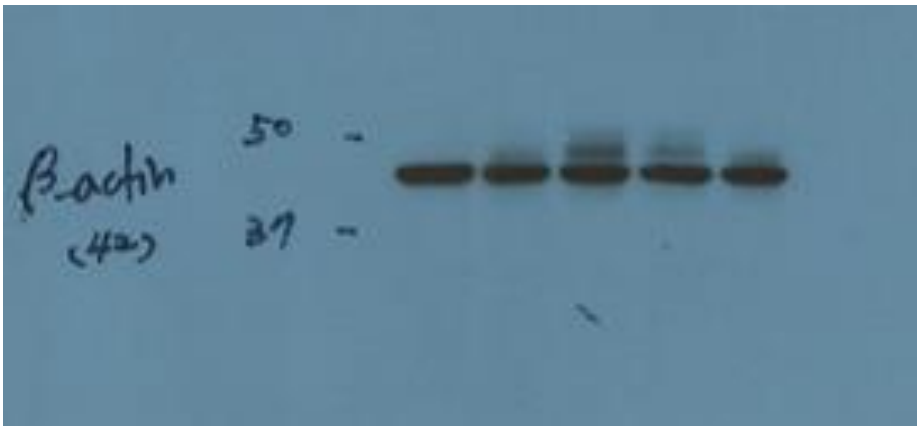

The original and unprocessed blot images of Figure 4D.

β-actin

PI

0 2 8 24 48

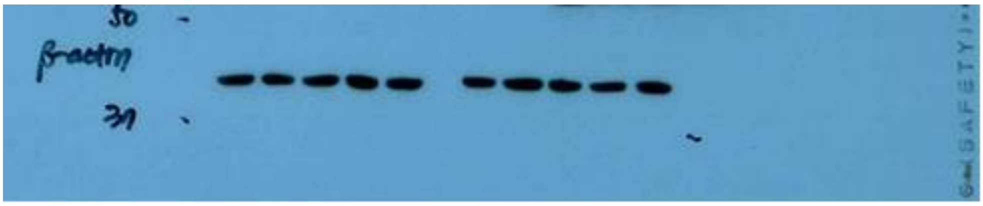

42kDa▶

IL-33

PI

0 2 8 24 48

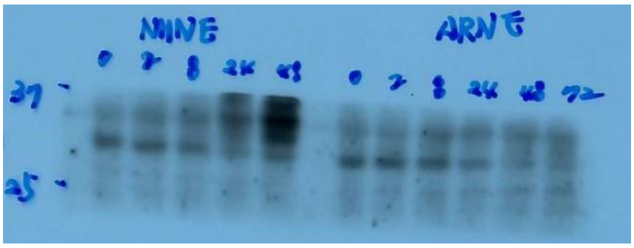

30kDa▶

RIPK3

PI

0 2 8 24 48

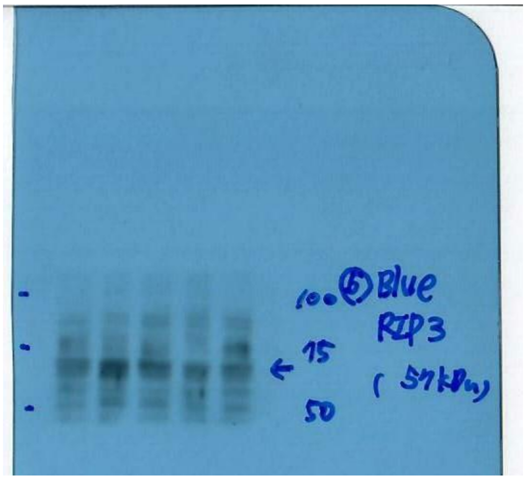

57kDa▶

pMLKL

PI

0 2 8 24 48

55kDa▶

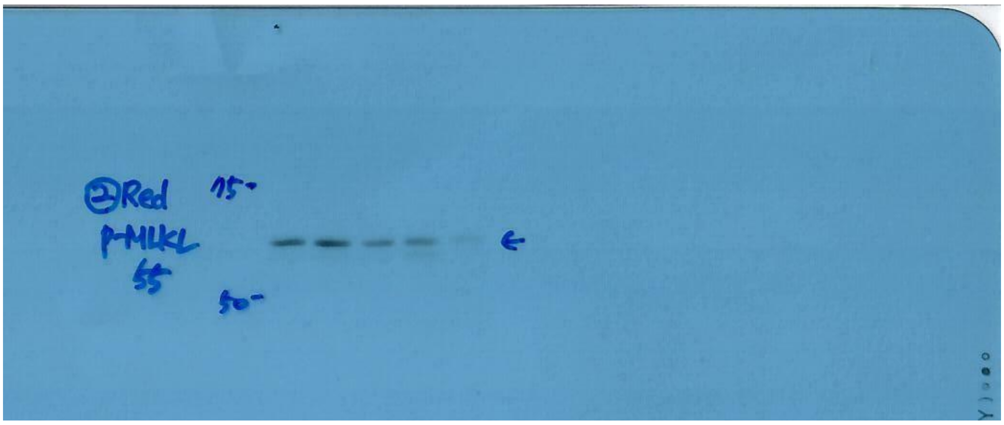

Total MLKL

PI

0 2 8 24 48

55kDa▶

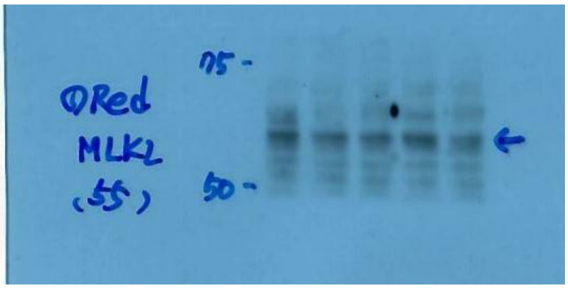

CASP3

PI

0 2 8 24 48

17kDa▶

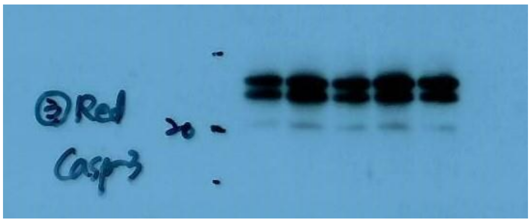

Supplement: Supplementary file 2 — Additional file 2: The original and unprocessed blot images Fig. 4C and D [file 12866_2023_2898_MOESM2_ESM.pdf]
